# The evolution of multicellularity and cell differentiation symposium: bridging evolutionary cell biology and computational modelling using emerging model systems

**DOI:** 10.1242/bio.061720

**Published:** 2024-10-07

**Authors:** Núria Ros-Rocher

**Affiliations:** Institut Pasteur, Université Paris-Cité, CNRS UMR3691, Evolutionary Cell Biology and Evolution of Morphogenesis Unit, 25-28 Rue du Dr. Roux, 75015 Paris, France

**Keywords:** Multicellularity and cell differentiation, Phenotypic plasticity, Eco-evo cell biology, Gene regulation, Modelling

## Abstract

‘The evolution of multicellularity and cell differentiation’ symposium, organized as part of the EuroEvoDevo 2024 meeting on June 25-28th in Helsinki (Finland), addressed recent advances on the molecular and mechanistic basis for the evolution of multicellularity and cell differentiation in eukaryotes. The symposium involved over 100 participants and brought together 10 speakers at diverse career stages. Talks covered various topics at the interface of developmental biology, evolutionary cell biology, comparative genomics, computational biology, and ecology using animal, protist, algal and mathematical models. This symposium offered a unique opportunity for interdisciplinary dialog among researchers working on different systems, especially in promoting collaborations and aligning strategies for studying emerging model species. Moreover, it fostered opportunities to promote early career researchers in the field and opened discussions of ongoing work and unpublished results. In this Meeting Review, we aim to promote the research, capture the spirit of the meeting, and present key topics discussed within this dynamic, growing and open community.

## INTRODUCTION

**Fig. 1.**
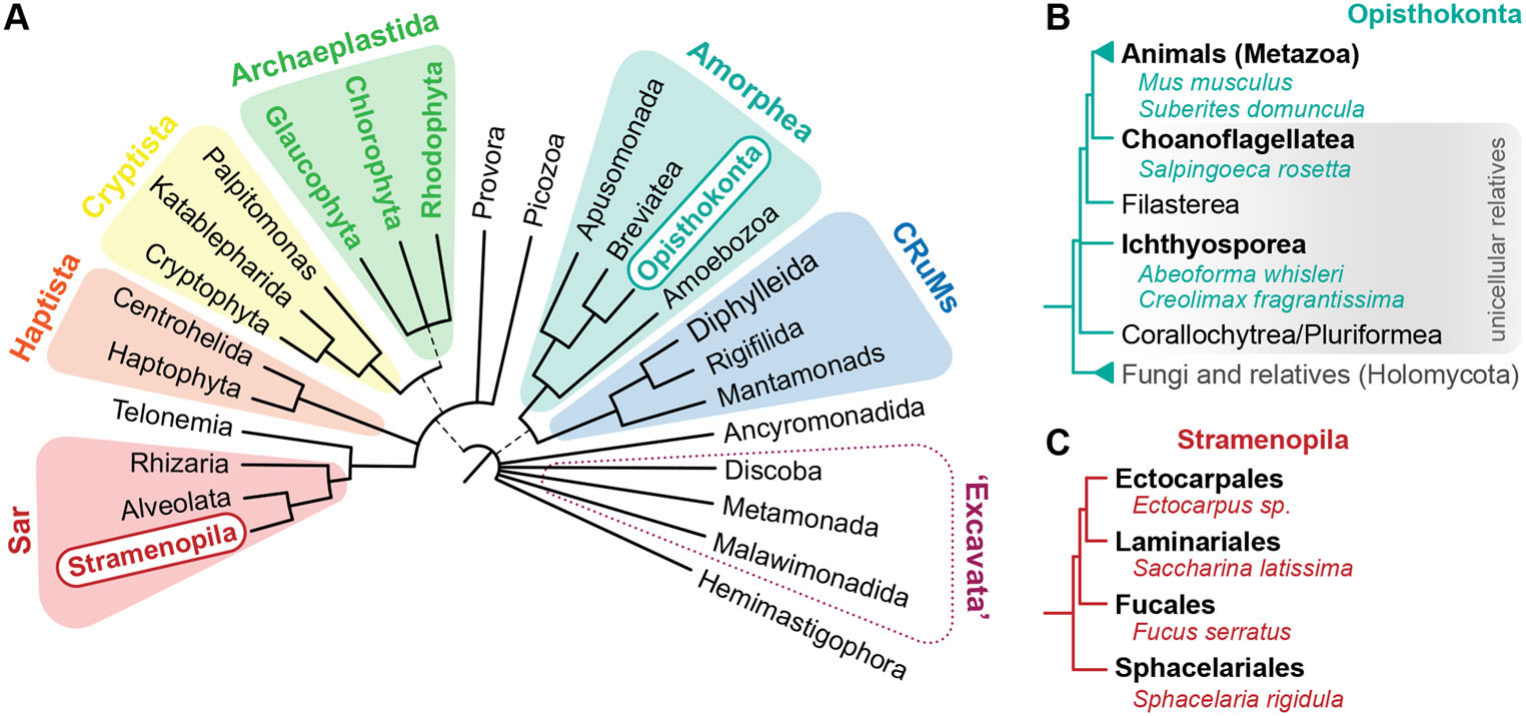
**The Eukaryotic tree of life and phylogenetic relationships within Opisthokonta and Stramenopila.** (A) The Eukaryotic tree of life, adapted from (Burki et al., 2020; Tikhonenkov et al., 2022). Colored groupings correspond to the currently recognized eukaryotic ‘supergroups’. Colored names within each supergroup represent lineages that evolved complex multicellularity in some of its members. The supergroup ‘Excavata’ is surrounded with a dotted line because of inconsistencies between the original supergroup definition (based on a distinctive morphology) and phylogenetic and phylogenomic studies (which later defined three monophyletic subgroups separated in distinct clades). Uncertain positions are represented with polytomies. Dashed lines reflect lesser uncertainties about the monophyly of certain groups. (B) Phylogenetic relationships of lineages within the Opisthokonta eukaryotic supergroup, showing the species used in the studies mentioned here. Topology based on (King et al., 2008; Fairclough et al., 2013; Grau-Bové et al., 2017; Torruella et al., 2015). Uncertain relationships are depicted as polytomies. (C) Simplified cladogram of brown algae, showing the species used in the studies mentioned here. Topology based on (Heesch et al., 2021).

Evolutionary transitions to multicellularity have occurred multiple times independently across the eukaryotic tree of life (Lamża, 2023). Compared to ‘simpler’ multicellular forms, ‘complex’ multicellular lineages (i.e. exhibiting controlled three-dimensional (3D) morphogenesis, a certain degree of cell–cell adhesion, and differentiated cells and tissues), like animals, plants and fungi, have evolved only a handful of times (Fig. 1A) (Knoll, 2011; Grosberg and Strathmann, 2007; Staps et al., 2019). Yet, the molecular and ecological basis for evolving cell differentiation and multicellularity is still a matter of debate, as is the question of whether the evolution of multicellularity follows similar paths in different lineages. In ‘The evolution of multicellularity and cell differentiation’ symposium, a diverse panel of experts discussed current and future approaches that can lead to significant advances in the field by combining experimental work, mathematical modelling and computational analyses using emerging model systems.

The symposium took place at the University of Helsinki City Centre Campus in the framework of the 9th Meeting of the European Evolutionary Developmental Biology Society (EuroEvoDevo 2024) on June 25-28th in Helsinki (Finland). It was co-organized by James M. Gahan (University of Galway, Ireland), Pawel Burkhardt (Michael Sars Center and University of Bergen, Norway) and Núria Ros-Rocher (Institut Pasteur, Paris, France). Here, we recapitulate the main topics and conclusions that were discussed during the symposium and provide a brief reflection on how the field could evolve in the future.

## Cell differentiation and behavioral switches in close animal relatives

How distinct cell types emerged and integrated in multicellular organisms remains a major evolutionary question. A current hypothesis proposes that cell differentiation could have emerged by the integration of pre-existing functionally different life stages of close unicellular ancestors of complex multicellular lineages, given that their extant unicellular relatives can differentiate into distinct life stages (reviewed in Brunet and King, 2017; Márquez-Zacarías et al., 2021; Ruiz-Trillo et al., 2023; Najle and Ruiz-Trillo, 2021). Here, four experts presented ongoing work using unicellular species closely related to animals as models and discussed the role of the environment in regulating cell differentiation and previously overlooked behavioral responses.

**Fig. 2.**
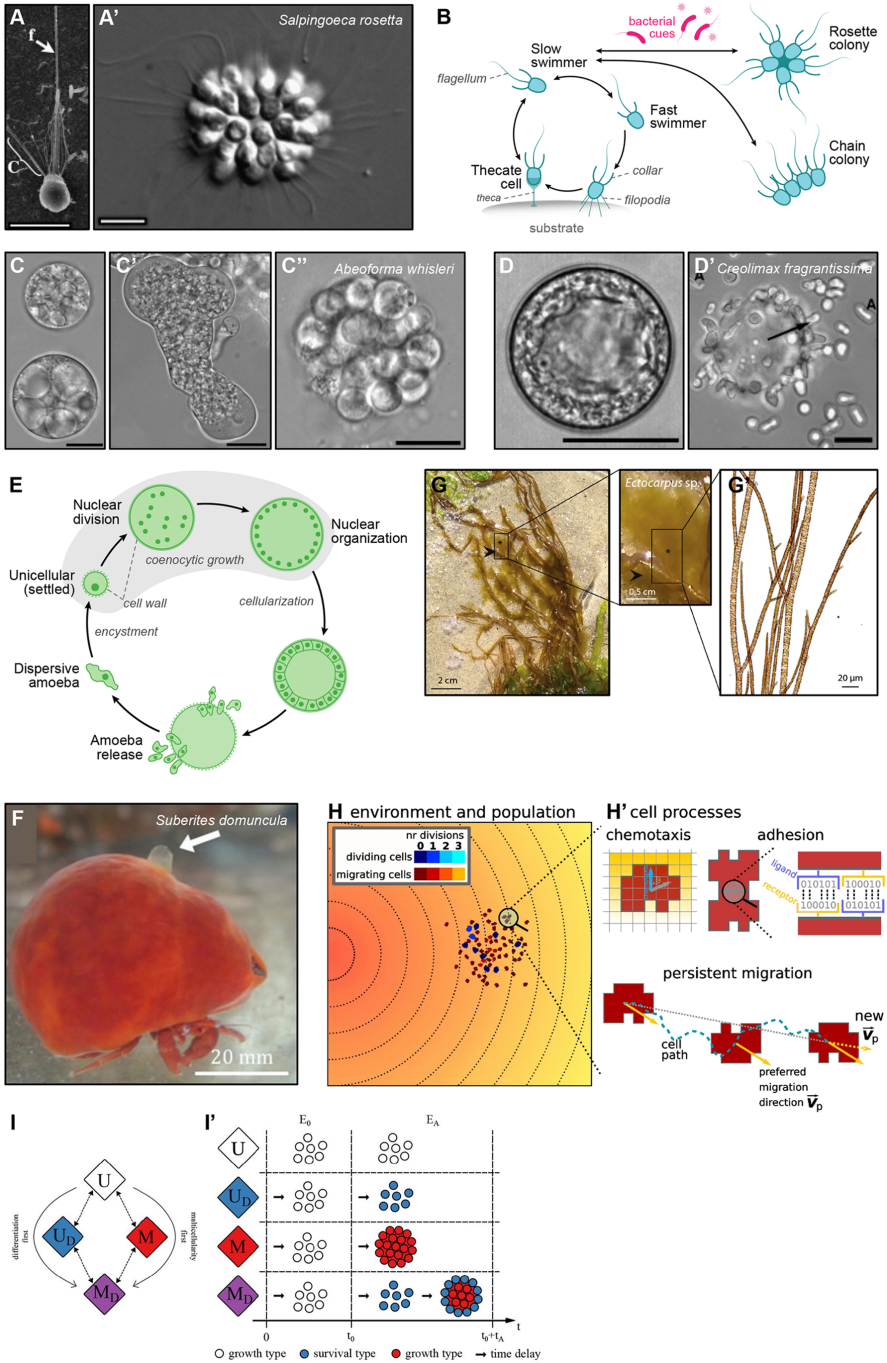
**Cell differentiation in close animal relatives and brown algae.** Cell morphology of the choanoflagellate *Salpingoeca rosetta* in its ‘slow swimmer’ cell stage (A) and multicellular ‘rosette’ colony (A′). The flagellum (f) and the collar (c) are defining features of choanoflagellate cells. Scale bars represent 5 µm. Images modified from (Dayel et al., 2011). (B) Life stages of *S. rosetta,* based on (Dayel et al., 2011). Single-celled ‘slow swimmers’ can transition to multicellular colonies (chains and rosettes) or to a sessile ‘thecate’ unicellular stage. Commonly observed cellular forms in the ichthyosporean *Abeoforma whisleri*. Cells can often be spherical with prominent vacuoles (C), transition to a plasmodial form (C′), and cellularize for reproduction during coenocytic division (C″). Scale bars: 20 µm. Images modified from (Marshall and Berbee, 2011). General morphology of *Creolimax fragrantissima*, depicting a spherical cell with a large central vacuole (D) and amoebae (indicated as ‘A’) escaping from a mature cellularized coenocyte (D’). Scale bars represent 20 µm. Images modified from (Marshall et al., 2008). (E) Life stages of the ichthyosporean *Creolimax fragrantissima* in standard growth conditions, based on (de Mendoza et al., 2015; Marshall et al., 2008). Single-nucleated amoebae settle and round the cell body onto a spherical cell that develops a cell wall. Rounded cells later undergo multiple rounds of nuclear division without cytoplasmic division (coenocytic growth). The coenocyte finally cellularizes and releases multiple amoebae, starting the cycle again. Image adapted from (Ros-Rocher et al., 2021; Najle and Ruiz-Trillo, 2021). Arrows in B and E indicate directionality of each life stage transition. (F) Sponge *Suberites domuncula* growing on a hermit crab shell. Image adapted from (Revilla-i-Domingo et al., 2018). (G) *Ectocarpus sp.* gametophyte (asterisk) in the field growing on the brown alga *Scytosiphon lomentaria* (arrowhead). (G′) Zoom-in of an *Ectocarpus sp.* gametophyte. Image adapted from (Coelho et al., 2020). (H) Model description in which individual cells in a given environment have to survive. The environment contains a chemoattractant gradient (lines and color indicate equal amounts of chemoattractant). (H′) Cells can sense the chemoattractant and move preferentially towards sites with higher chemoattractant concentration (blue arrow). Adhesion between two cells is mediated by receptors and ligands. Persistent migration is implemented by endowing each cell with a preferred direction of motion *v_p_*. Image modified from (Vroomans and Colizzi, 2023). (I) Model schematics showing different evolutionary routes from unicellularity (U) to differentiated multicellularity (M_D_), including intermediate stages depending on whether unicellular populations evolve differentiation or multicellularity first. (I′) Schematics of different evolutionary routes taken by unicellular populations in response to environments without stress (E_0_) or with an abiotic stress (E_A_). Image modified from (Isaksson et al, 2024).

Animals (Metazoa) are closely related to diverse lineages of unicellular species (Fig. 1B), which can differentiate into distinct cell stages (including multicellular stages) in laboratory cultures and presumably in their natural habitat (Sebé-Pedrós et al., 2013; Brunet et al., 2019; Hehenberger et al., 2017; Dayel et al., 2011; Marshall and Berbee, 2011; Marshall et al., 2008; Dudin et al., 2019; Leadbeater, 2015; Tikhonenkov et al., 2020; Kożyczkowska et al., 2021). Choanoflagellates (Fig. 1B), the sister group to animals, are bacterivorous aquatic microeukaryotes that display morphologically distinct cell types and an extensive ‘sensory molecular toolkit’ (Ros-Rocher and Brunet, 2023; Leadbeater, 2015). The marine species *Salpingoeca rosetta,* the best-studied choanoflagellate model, transitions between unicellular and multicellular stages which can take the form of chain colonies or bacterially-induced rosettes (Fig. 2A-B) (Booth and King, 2022; Dayel et al., 2011). Jeffrey Colgren (Michael Sars Center, University of Bergen, Norway), presented a novel cellular behavior associated with calcium signaling in *S. rosetta* in both unicellular and multicellular stages (Colgren and Burkhardt, 2024 preprint). Due to the recent development of genetic tools in this species (Booth et al., 2018; Booth and King, 2020), Jeffrey established a stable cell line expressing the calcium indicator RGECO1 and used this to describe calcium dynamics in *S. rosetta*. Remarkably, *S. rosetta* exhibited a range of spontaneous calcium transients, some with similar dynamics to excitable cell types in animals (Wan and Jékely, 2021; Leys, 1999, 2015; Leys et al., 2007), which turned out to be dependent on calcium entry from the extracellular environment. Jeffrey showed that some of those transients relied on voltage-gated calcium channels (VGCC), showcasing an associated cellular behavior consisting of rapid apicobasal contractions of the cell and flagellar arrest. Surprisingly, in *S. rosetta* chains and rosettes, calcium-mediated electrical signaling resulted in asynchronous and synchronous events, respectively, suggesting regulated communication between cells of a colony. The ciliary arrest coupled with apicobasal cell contraction was also synchronized with signaling, resulting in coordinated behavior. These findings highlight the role of calcium signaling for cell–cell communication and coordinated behavior in the multicellular colonies of a close relative of animals.

Choanoflagellates are also relevant to key ecological processes in aquatic environments, such as nutrient cycling. David Booth (UCSF, USA), presented the work of PhD student Fredrick Leon and colleagues, showing another case of cell differentiation linked to environmental nutrient cycles in *S. rosetta*. In a recent preprint, they reported that *S. rosetta* can assimilate iron from insoluble ferric colloids more efficiently through increased expression of the *S. rosetta cytochrome b561* iron reductase (cytb561a), a DCYTB ortholog (Leon et al., 2024 preprint). *S. rosetta* in their solitary sessile form (or “thecate cells”, Fig. 2B) display a different transcriptional profile than other unicellular and multicellular stages, upregulating many genes associated with signal transduction, gene regulation and nutrient acquisition. Notably, the *cytb561a* iron reductase was among the most highly expressed genes in thecates, which turned out to be the only cell stage expressing *cytb561a* at a high level regardless of the abundance or type of environmentally available iron. This cell-type-specific expression highlights cell differentiation as a strategy to provide a significant advantage in nutrient-poor environments, in this case by enabling the uptake and use of insoluble iron for increased cell proliferation. David further commented on the broader implications of these findings for understanding the evolutionary adaptations of choanoflagellates given the nutrient cycles in marine ecosystems. Iron is an essential limiting micronutrient in the oceans, which recirculates from the deep ocean to the surface by upwelling. David speculated that choanoflagellates likely play a crucial role in iron cycling within marine ecosystems, potentially by redirecting nutrient flow in microeukaryotic communities in the oceans. In fact, data analysis from the Ocean Gene Atlas metagenomic database showed that the abundance of choanoflagellate *cytb561a* transcripts correlated with upwellings (Leon et al., 2024 preprint). This study expands our understanding on how the environment impacts cell differentiation in a choanoflagellate, highlighting an important adaptation that likely influenced the evolution of nutrient acquisition strategies in early animal ancestors.

Other animal relatives may harbor additional undiscovered morphological diversity hidden in the environment. In Ichthyosporea (Fig. 1B), a clade composed mostly of animal symbionts and parasites, many species develop through multinucleate coenocytes and differentiate into distinct life stages under culture conditions (reviewed in Shabardina et al., 2024). In the last decade, efforts have focused on generating omic resources (Grau-Bové et al., 2017; Torruella et al., 2015; Dudin et al., 2019; de Mendoza et al., 2015) and developing genetic tools (Faktorová et al., 2020; Suga and Ruiz-Trillo, 2013) for a still-limited number of species. Given the difficulty of culturing many ichthyosporean species and, in some cases, the cryptic nature of their frequent habitat as animal parasites, little is known about the conditions that favor differentiation into distinct cell stages and their full morphological diversity. Elena Casacuberta (Multicellgenome Lab, Institute of Evolutionary Biology, CSIC-Universitat Pompeu Fabra, Spain) presented the work of postdoctoral researcher Victoria Shabardina and PhD student Fernando Bascón showing the potential morphological plasticity in the ichthyosporean *Abeoforma whisleri* (Fig. 2C) (Marshall and Berbee, 2011)*.* Their work in progress characterizes a previously unnoticed *A. whisleri* cell stage reminiscent of certain animal cell types (Shabardina et al., unpublished). Patricia Suárez Ara, a postdoctoral researcher in the same lab, presented another case of phenotypic plasticity in the ichthyosporean *Creolimax fragrantissima* (Fig. 2D-E) (Marshall et al., 2008)*.* In her work led by Sebastián R. Najle (Center of Genomic Regulation, Spain), Patricia showed an alternative *C. fragrantissima* life cycle in nutrient-limiting conditions with different nitrogen sources (Suárez Ara, 2024). Patricia and colleagues are currently characterizing the phenotypical and transcriptional response of *C. fragrantissima* upon nitrogen deprivation to unravel the molecular determinants of this phenotypic change (Suárez Ara et al., unpublished). Both talks stressed the importance of deeper morphological observations in these species to unravel the cellular and molecular determinants of cell differentiation into novel cell types.

Altogether, these experts highlighted close unicellular relatives of animals as emerging models for understanding phenotypic plasticity and cell differentiation in their ecological context. We expect future work to explore how protists regulate their life cycle transitions and integrate environmental signals, ultimately enhancing our understanding of the evolutionary and ecological foundations of cell differentiation and multicellularity in the animal stem.

## The regulatory and molecular determinants of cell differentiation and multicellularity in animals and brown algae

In multicellular organisms, like animals and brown algae, the progression from a single cell to a multicellular organism relies on defined gene expression programs, which notably differ between distinct cell types. The following panel discussed recent advances in understanding the regulatory strategies used during animal and brown algae development from a molecular and a cell biology perspective.

Alex de Mendoza (Queen Mary University of London, UK), focuses on the evolution of gene regulation in various eukaryotic lineages. Alex presented a case of surprising conservation in Sox and POU stem cell-associated transcription factors (TFs), previously thought to be animal-specific (Gao et al., unpublished). In collaboration with Ralf Jauch's team in Hong Kong University, they discovered members of both TFs in the genomes and transcriptomes of various unicellular animal relatives, including choanoflagellates. Notably, the choanoflagellate Sox TF shares the same TF binding motif as the mammalian Sox, and heterologous expression of the choanoflagellate Sox in mouse proved it also binds mouse DNA like mammalian Sox2. Overexpression of the choanoflagellate Sox in mouse cells can also induce pluripotent stem cells (iPSC) capable of incorporating into mouse embryos, leading to chimeric mice. In contrast, the choanoflagellate POU, which displays a different amino acid change compared to mammalian POU, binds DNA differently than animal POUs and cannot induce pluripotency, as it retains an ancestral Homeobox motif preference. Alex finally discussed how critical changes in TFs DNA-binding specificity and their interactomes from a pre-existing set of Sox and POU might have led to a stepwise formation of the Sox-POU animal regulatory network.

Roger Revilla-i-Domingo (University of Vienna, Austria), also investigates the gene regulatory mechanisms underlying stem cell differentiation in early animal evolution. Roger's team studies *Suberites domuncula,* a sea sponge that colonizes gastropod shells occupied by hermit crabs (Fig. 2F). Similar to other sponges, *S. domuncula* explants are capable of stem cell-mediated regeneration into functional sponges (Revilla-i-Domingo et al., 2018). Roger's team provided a complete genome assembly, single-cell transcriptomics (scRNAseq), chromatin accessibility profiling and a transfection tool (Revilla-i-Domingo et al., 2018) to reveal cell differentiation trajectories in *S. domuncula,* turning it into a new experimentally tractable model system. Notably, scRNAseq of regenerating sponge tissue revealed a Myc TF homolog, key for cell proliferation and differentiation in mice (Wilson et al., 2004; Li-Bao et al., 2024), as a candidate for sponge regeneration. Roger presented the work of PhD student Carolina Elisabeth Atria (co-supervised with Claudia Plant) and colleagues, where they inferred potential Myc targets in *S. domuncula* based on expression profile similarity and Myc-DNA binding motif analyses. The Myc-DNA interactions were further explored using AlphaFold3 and revealed 51 direct targets in *S. domuncula*, which, based on comparison with published Myc targets in mouse, might be ancestral direct targets of Myc in stem cell differentiation. Ongoing work focuses on experimentally validating candidate target genes inferred in *S. domuncula* through established transgenesis methods, which will offer key insights into the mechanisms regulating emergence of specialized cell types in early animal evolution. In future work, his team will combine single cell transcriptomics and chromatin profiling approaches in unicellular relatives of animals to further infer putative regulatory changes at the onset of animals.

Brown algae (Stramenopila) represent another major lineage of exclusively multicellular organisms, having evolved multicellularity independently of other eukaryotes over a billion years ago (Fig. 1A) (Bringloe et al., 2020). Like animals, brown algae are notable for their remarkable morphological and structural diversity, which arises from the differentiation of specialized cells into tissues and organs through tightly regulated morphogenetic programs (Charrier et al., 2008, 2012). Liping Wang (Max Plank Institute for Biology, Germany), uses the brown algal model *Ectocarpus* to unravel the regulatory networks and functional modules of multicellular development (Figs 1C and 2G) (Coelho, 2024; Coelho et al., 2020). Liping and colleagues in the Coelho group are using single-nuclei RNA sequencing (snRNAseq) to generate a comprehensive cell-type-specific expression atlas in *Ectocarpus*, which allows them to distinguish transcriptionally and morphologically distinct cell types (unpublished). *Ectocarpus* is also a historical model for DNA virus and host interactions, which are known to impact algal reproduction (Charrier et al., 2008; 2012; Müller, 1964; Batista et al., 2024). The group is currently exploring how *Ectocarpus* and a giant dsDNA endogenous virus, the *Ectocarpus siliculosus Virus* (EsV-1), tightly coordinate their life cycles. In particular, building on recently developed snRNAseq approaches, Liping is studying how viral replication is activated in specific cell types of the host, and she expects to illuminate the molecular determinants underlying the dynamics of virus/algae interaction.

Finally, Marie Zilliox (Functional Genomics Institute in Lyon, CNRS-ENS, France), studies brown algae from a cell biological perspective to find the determinants of 3D growth during development. Animal cells rely on cell migration to complete morphogenesis in 3D, yet brown algae cells rely on other strategies, given their semi-rigid cell walls (Charrier et al., 2012). To shed light on this process, Marie developed an *in vivo* monitoring set-up using fluorescence live imaging and an *ad-hoc* image analysis pipeline (Zilliox *et. al.,* unpublished). By comparing three different brown algae species (*Sphacelaria rigidula, Fucus serratus* and *Saccharina latissima;* see Fig. 1C) Marie confirmed the cell division patterns in 3D live imaging. For instance, *S. rigidula* apical cells grow along a single axis and then sub-apical cells divide to grow in other dimensions. *S. latissima* grows a monolayer of a thousand cells before thickening. Finally, *F. serratus* initiates cell division without cell expansion, a process reminiscent of early embryo segmentation in animals. In ongoing work, Marie aims to uncover the mechanisms determining the timing, orientation and position of cell growth in 3D during algae embryogenesis.

## Modelling the evolution of multicellularity and cell differentiation

Another important theme of the symposium was the use of computational and mathematical models to simulate the evolution of multicellularity and cell differentiation (Rossetti et al., 2010). These simulations can integrate knowledge from biological phenomena, and draw inspiration from microbes and simple multicellular organisms (Schaap, 2011; Kaiser, 2003; Smith et al., 2019), as well as diverse features (e.g. cell–cell adhesion and germ–soma differentiation; see Knoll, 2011; Ratcliff et al., 2012) and processes (e.g. embryogenesis and cancer; see Weijer, 2009; Friedl and Gilmour, 2009) in obligate multicellular organisms. Here, two experts modelled how cells interact with one another and with the environment and assessed how they adapt to distinct selective pressures by evolving novel traits and collective behaviors.

Renske Vroomans (Sainsbury Laboratory, University of Cambridge, UK) showed a simple model where individual cells, featuring certain default levels of adhesion, had to migrate up chemoattractant gradients towards resources for survival and reproduction (Fig. 2H). Notably, cells evolved their adhesion to other cells depending on the distribution of resources (e.g. nutrients) in the environment, as observed in Dictyostelids and Myxobacteria (Du et al., 2015; Kaiser et al., 1979). In an environment with sufficient resources, cells evolved increased adhesiveness and formed multicellular aggregates, which located resources more efficiently than single cells by emergent collective chemotaxis. However, in resource-limited environments where even clusters of cells could not locate resources, cells maintained low adhesiveness to enhance dispersal (Colizzi et al., 2022; 2020; Vroomans and Colizzi, 2023). In a follow-up model, Renske included the evolution of the cellular gene regulatory machinery, which underwent a strong repurposing to evolve multicellularity. Notably, cells that previously prioritized survival by migrating to resources, evolved to exploit the efficient cluster migration, shifting their priority to reproduction (Vroomans and Colizzi, 2023). Thus, different environments determined the competition dynamics between cells and resulted in different unicellular or multicellular evolutionary paths. Renske's team is currently extending the model further to investigate the evolution of complex cell behavior at the onset of multicellularity.

The acquisition of emergent behaviors, including chemotaxis, polarity, and multicellularity, can also be modelled in fluctuating environments, where cells need to sense, respond, and adapt to changing signals over time. Eric Libby (IceLab, Umeå University, Sweden), discussed how different evolutionary paths can lead to multicellularity (including differentiated multicellularity) by modelling the response of different populations to fluctuations between environments with and without stress (Fig. 2I) (Isaksson et al., 2024 preprint). In this model, unicellular populations used different routes to evolve differentiated multicellularity upon abiotic stress depending on whether they first evolved differentiation (survival phenotype) or multicellularity (growth phenotype). Both phenotypes could be combined in a joint multicellular differentiated lifestyle for increased survival that shielded reproductive cells in the center of a multicellular group. Although unicellular populations could evolve differentiated multicellularity, Eric showed that they frequently reverted back to unicellularity after further adaptation. Effectively, new beneficial mutations (particularly ones increasing growth rate or survival) rendered multicellularity and/or differentiation superfluous. Based on these results, Eric discussed the role of chance in determining whether populations in the model fixed complex multicellularity or ultimately reverted to unicellularity.

Together, both talks demonstrated that eco-evo-devo computational models can be useful in studying the evolution of fitness-relevant multicellular traits. Unicellular organisms exposed to environmental fluctuations and limiting resources follow various adaptive strategies to gain and/or lose complexity, including re-purposing their regulatory adhesion and fate determination toolkits. This, together with the interactions with a changing environment, can lead to distinct evolutionary paths to improved survival by evolving multicellularity, cell differentiation, or a combination of both. In the coming years, we expect new modelling work to bridge theoretical concepts with empirical biological phenomena, providing crucial insights into the evolution of cell types and multicellularity.

## Conclusions

This symposium brought together experts with complementary expertise to address the evolution of multicellularity and cell differentiation from different perspectives. The field is experiencing significant growth as collaborative efforts are increasing available omic resources and refining the eukaryotic phylogenetic framework for comparative studies. The community is also expanding the range of experimentally tractable organisms from diverse lineages to functionally address these questions. Efforts are also directed in improving mathematical and computational models to complement experimental studies and illustrate mechanisms (and broader principles) for the evolution of novel traits. We anticipate significant progress in the field thanks to joint efforts in developing phylogenetically relevant non-model species with unique biological and ecological features for comparative studies. This symposium sparked dynamic discussions between students, early-career researchers, and senior leaders, fostered networking and forged new collaborations, setting the stage for future breakthroughs.
